# From Scourge to Cure: Tumour-Selective Viral Pathogenesis as a New Strategy against Cancer

**DOI:** 10.1371/journal.ppat.1003836

**Published:** 2014-01-16

**Authors:** Carolina S. Ilkow, Stephanie L. Swift, John C. Bell, Jean-Simon Diallo

**Affiliations:** 1 Centre for Innovative Cancer Therapeutics, Ottawa Health Research Institute, Ottawa, Ontario, Canada; 2 Swift Science Writing, Hamilton, Ontario, Canada; 3 Department of Biochemistry, Microbiology and Immunology, University of Ottawa, Ottawa, Ontario, Canada; University of Alberta, Canada

## Abstract

Tumour mutations corrupt cellular pathways, and accumulate to disrupt, dysregulate, and ultimately avoid mechanisms of cellular control. Yet the very changes that tumour cells undergo to secure their own growth success also render them susceptible to viral infection. Enhanced availability of surface receptors, disruption of antiviral sensing, elevated metabolic activity, disengagement of cell cycle controls, hyperactivation of mitogenic pathways, and apoptotic avoidance all render the malignant cell environment highly supportive to viral replication. The therapeutic use of *oncolytic viruses* (OVs) with a natural tropism for infecting and subsequently lysing tumour cells is a rapidly progressing area of cancer research. While many OVs exhibit an inherent degree of tropism for transformed cells, this can be further promoted through pharmacological interventions and/or the introduction of viral mutations that generate recombinant oncolytic viruses adapted to successfully replicate only in a malignant cellular environment. Such adaptations that augment OV tumour selectivity are already improving the therapeutic outlook for cancer, and there remains tremendous untapped potential for further innovation.

## The Tumour: A Unique Niche for Virus Growth

**Figure 1:**
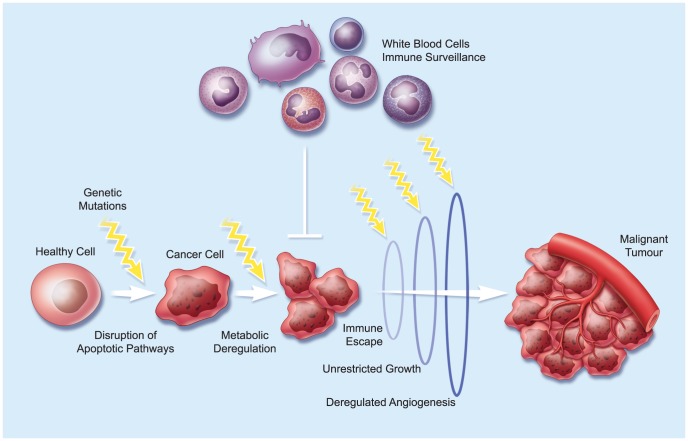
Tumour Evolution. A hypothetical pathway of tumour evolution from a normal cell to an advanced-stage cancer. Mutations in key regulatory genes lead to changes in cell physiology that favour tumour growth. Over time, these genetic defects accumulate to confer many of the known hallmarks of cancer [131]. The sequence of these events and the timing represented here is only one example of how this might occur.

Tumour progression is generally considered a stochastic process, but is nevertheless associated with a series of hallmark changes that include, among others, resistance to apoptosis, metabolic deregulation, immune escape, growth independence, and enhanced angiogenic capacity [1] (Figure 1). Either taking certain pathways offline, or boosting their activity, disrupts cellular homeostasis and creates a supportive environment that facilitates exponential cancer cell growth. While such changes allow the malignant cell a competitive survival and growth advantage over “normal” cells, they also render it susceptible to infection, since many of the pathways subverted by the tumour are also necessary for effective antiviral responses.

Once a virus penetrates a tumour cell, the malignant metabolic infrastructure provides abundant support for viral replication. Since the same pathways that are already boosted during cancer cell transformation are also engaged by viral replication, tumour cells are attractive targets for OVs, a class of cancer biotherapeutics that includes such diverse virus families as rhabdoviridae (e.g., vesicular stomatitis virus [VSV], Maraba virus), poxviridae (e.g., vaccinia [VV], myxoma [MYXV]), adenoviridae (e.g., adenovirus serotype 5 [Ad5], Colo-Ad1), paramyxoviridae (e.g., Newcastle disease virus [NDV], measles virus [MV]), togaviridae (e.g., Sindbis virus [SV]), herpesviridae (e.g., herpes simplex virus-1 [HSV-1]), reoviridae (e.g., reovirus type III), picornaviridae (e.g., poliovirus, coxsackievirus), and parvoviridae (e.g., H1-parvovirus). The current status of clinical trials for such therapeutic OVs has recently been reviewed by Russell and colleagues [2].

In this review, we highlight the similarities between the requirements for optimal cancer cell growth and successful viral replication, and the mounting evidence that tumours with altered metabolic and signaling networks provide a unique niche for OV propagation. While some viruses are inherently oncophilic, it is also possible to direct selective growth in cancer cells by inactivating or deleting certain viral virulence genes whose lost functions are complemented by mechanisms that drive malignancies and distinguish tumours from normal healthy tissues. The application of such genetic interventions and/or the potential for coadministering pharmacological compounds to enhance OV activity specifically in tumours will be discussed.

## Overexpressed Tumour Antigens: Entangling Viruses

**Figure 2:**
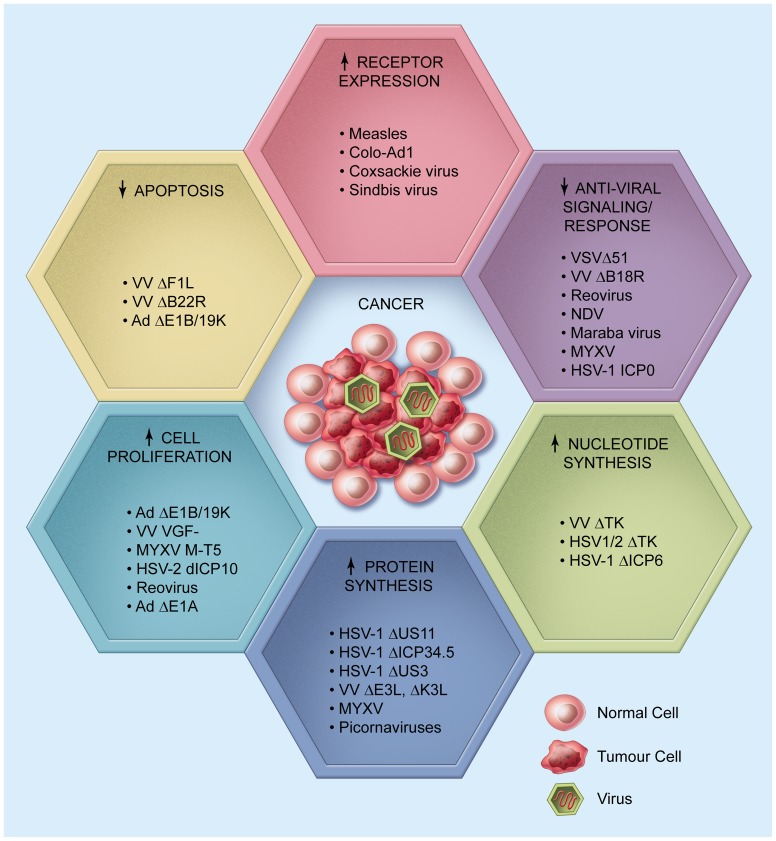
Oncolytic Viruses Are Designed to Grow in the Tumour Niche. There are at least six key critical features of tumour cell growth that can be targeted by oncolytic viruses. These include changes in the expression of viral-host cell receptors, the antiviral response, nucleotide and protein synthesis, cell proliferation, and apoptosis. A number of engineered or selected oncolytic viruses exist that can exploit one or more of these malignant characteristics.

Host cell entry is one of the first challenges that viruses must overcome to access intracellular replication sites, and the availability of cell surface receptors for viral attachment and uptake is of paramount importance. Malignant cells undergo tremendous changes in the profile of cell surface receptors they display, and the tumour specificity of many oncolytic viruses often begins with engaging these overexpressed antigens at the cell surface (Figure 2). For example, poliovirus binds the cell surface receptor, nectin-like molecule 5 (NECL-5) [3], which is expressed at very low levels in normal tissues, but is broadly overexpressed in several solid tumours and in the proliferating vasculature that supports them, including glioblastoma multiforme and ovarian, prostate, colorectal, and lung carcinomas [4]–[8]. This natural biological observation has been exploited to create a potent chimeric OV that selectively targets a variety of tumours of neuroectodermal origin [9]. Similarly, measles virus (MV) and the chimeric adenovirus ColoAd1 both bind the cell surface receptor, CD46, which is commonly upregulated by cancer cells [10],[11]. MV gene expression, cytopathic effect, and oncolysis have all been correlated with density of CD46 on the cell surface [12]. Nectin-4 can also be exploited for MV entry [13], and is highly expressed in lung, breast, colon, and ovarian carcinomas [14]–[16]. The natural oncolytic capacity of coxsackievirus A21 (CVA21) is based on high expression of intracellular adhesion molecule 1 (ICAM-1) and/or decay acceleration factor (DAF) on the surface of malignant cells [17], while the uptake of coxsackievirus B3 (CVB3) and certain oncolytic adenoviruses is particularly enhanced in medulloblastoma, neuroblastoma, and endometrial carcinomas overexpressing the host cell coxsackievirus–adenovirus receptor (CAR) [18],[19]. Oncolytic Sindbis virus (SV) binds the Laminin receptor (LamR) [20], which is modestly expressed in almost every mammalian cell, but highly upregulated in many solid tumours [21]–[24]. In normal cells, LamRs are occupied by the laminin ligand and SV attachment is outcompeted, while in cancer cells, the overexpression of LamRs exposes a significant number of unoccupied SV target sites [25].

OVs have also been reprogrammed to bind specific tumour surface antigens through the display of single-chain antibodies or polypeptide-binding ligands on the virion surface, or by fusing scaffolding moieties within viral surface proteins (reviewed in [26]). For example, several ligands have been successfully displayed on the surface of MV particles, including single-chain antibodies against EGFR [27],[28], CD20 [29], CD38 [27], and the folate receptor alpha [30]. Similarly, herpes simplex virus (HSV) and various adenoviruses have been genetically redirected toward tumours through the incorporation of cancer-specific receptor binding ligands into viral surface proteins [31]–[35]. Finally, increased oncotropism of VV has been achieved by fusing targeting moieties against the Human Epidermal Growth Factor Receptor 2 or Mucin-1 to virus envelope resident proteins [36],[37].

## A Dysfunctional Cellular Antiviral Defense: The Achille's Heel of Cancer

An estimated 65–70% of cancer cell lines have defective interferon (IFN) responses [38]. However, both the cancerous and stromal compartments of a tumour integrate antiviral signals to establish a scale of IFN responsiveness, which is different for each malignancy. The biological drivers of cancer evolution that lead to the loss of interferon responsiveness are not completely understood, but it is thought that physiological processes that favour antiviral activity are incompatible with efficient tumour growth. For instance, interferon and interferon-responsive genes are anti-angiogenic [39], and are known to induce apoptosis [40], cell growth arrest [41], and immune stimulation [42], all of which cancer cells strive to evade. Not surprisingly, one of the most common genetic changes in the tumour as it transitions to a stealth phenotype is the loss of expression of genes from the interferon pathway, and the establishment of further rounds of *immune editing* that allow the cancer to become invisible to the host immune system [43]. As tumours evolve to full malignancy, this elimination or inactivation of certain interferon gene products may allow a growth advantage, but also compromises cellular antiviral responses to varying degrees.

The IFN pathway is part of the innate immune response triggered upon pathogen entry by a limited number of cellular immune receptors recognizing broadly conserved pathogen-associated molecular patterns (PAMPs). Most wild-type viruses encode gene products that antagonize interferon response signaling, and can robustly infect tumour and normal cells alike. Some “natural” OVs have evolved to infect nonmammalian organisms (e.g., Newcastle disease virus (NDV) in avian hosts) and thereby lack virulence genes that can effectively antagonize mammalian antiviral responses; such viruses only prosper in tumour cells that acquire mutations in interferon response genes [44]. For other viruses, it is necessary to engineer defects in virulence gene products that normally antagonize interferon responses to confer a robust growth response in cancer cells but an inability to productively infect normal tissues [45]–[47] (Figure 2).

The recognition that interferon signaling is in large part critical to OV selectivity made it clear that manipulation of this cellular response could be a target for therapeutic intervention. Tumour cells that have completely lost their interferon response provide an ideal substrate for OV replication. Such tumours can easily be cured with a “single shot” of virus [48]. However, most human tumours are genetically and architecturally heterogeneous, and thus the extent of their antiviral responsiveness can be variable and provide a significant barrier to OV therapy [49]. This problem can be biologically addressed by enhancing the potency of existing OVs [50],[51], selecting inherently more potent virus backbones (such as Maraba, which outperforms several related OVs [e.g. VSV] in terms of replication speed, viral productivity, and lytic capacity across multiple cancer cell lines [52]), or coinfecting tumours with distinct attenuated OVs that can complement each other's growth in a hostile tumour environment [53]. Coinfection of tumours with an oncolytic vaccinia virus (VV) that expresses the IFN-scavenging protein, B18R, can significantly enhance the ability of attenuated, interferon-sensitive vesicular stomatitis virus (VSV) to spread through tumours exhibiting partial defects in their antiviral defenses, resulting in improved oncolytic activity compared to either virus alone [53].

Several pharmacological strategies have aimed to improve OV therapy by disrupting IFN responses in resistant tumour cells. Our group, and others, have previously shown that histone deacetylase inhibitors (HDIs) can improve the oncolytic activity of OVs both *in vitro* and *in vivo*, including VSVΔ51, several strains of VV, and herpes simplex virus (HSV) [54]–[56]. HDIs profoundly impact cellular epigenetics, and inhibit the IFN response by blocking the transcriptional upregulation of IFN-stimulated genes following viral infection or IFN signalling [57],[58]. A related strategy has been to coadminister small molecules, selected on the basis of their ability to enhance viral oncolysis. Using this method, we recently identified a class of *viral sensitizer* compounds (VSe1–15) specifically selected for their superior activity in comparison with the clinically approved HDI, Vorinostat. Such viral sensitizers robustly enhance VSVΔ51 growth in IFN-resistant tumour cells [59]. Importantly, the “proviral” effects of both HDIs and novel viral sensitizers only occur in tumour cells, which may be attributable to the tumour microenvironment already being conducive to viral growth.

## Dysregulated Tumour Metabolism: Fueling the OV Fire

### Exploiting Increased Nucleotide Pools

As tumours expand and develop, metabolic activity on a per-cell basis increases to sustain cellular output and fuel biosynthetic processes. Many cancer cells meet this demand by reprogramming energy production from mitochondrial phosphorylation to aerobic glycolysis, a phenomenon known as the “Warburg effect” [60]. Since efficient virus replication and virion assembly is a similarly energy demanding process fueled by the host cell, certain viruses may act to skew cellular metabolism toward glycolysis to divert glycolytic intermediates into biomachinery programs that synthesize viral macromolecules. For example, the VV *N1L* gene encodes a multifunctional protein that targets different cellular kinases, one of which influences ATP levels during virus replication [61].

While a metabolic boost increases overall levels of biomolecules in host cells, several viruses also directly impact the size of these biosynthetic pools. Large DNA viruses achieve replicative self-sufficiency by encoding enzymes involved in the *de novo* synthesis of deoxynucleotides (dNTPs). For example, herpes- and orthopoxviruses encode both large (R1) and small (R2) subunits of ribonucleotide reductase (RR), a key enzyme involved in catalyzing the conversion of ribonucleotides to dNTPs [62]. Other oncolytic poxviruses encode at least one RR subunit. In the absence of virally supplied RR proteins, successful virus replication depends on host cell RR activity, which is reportedly enhanced in malignant cells [63] and acts together with elevated levels of dNTPs to enable viral tumour selectivity. Selective deletions in viral genes encoding RR subunits serve to enhance the oncotropism of OVs. For example, HSV-1 and HSV-2 mutants with deletions in the *ICP6* gene, which encodes the large subunit of RR, often preferentially replicate in actively dividing cells [64], although some reports have shown replication in quiescent cells with mutations in *p16* [65]. Herpes- and poxviruses also encode the enzyme thymidine kinase (TK), which is primarily responsible for phosphorylating thymidine, a key step in DNA synthesis. Deletion or inactivation of TK in VV or HSV-1 leads to preferential viral replication in host cells with high levels of TK, such as tumour cells (Figure 2). Inactivating viral products involved in nucleotide metabolism has been a widely utilized and successful strategy to generate tumour-selective viruses: two of the top three current clinical OV candidates, OncoVEX GM-CSF and JX-594, contain such deletions and/or mutations [66],[67].

Relatively few attempts have been made to pharmacologically exploit the nucleotide synthesis pathway and simultaneously address the issue of tumour heterogeneity for OV growth. One example comes from Passer *et al.* [68], who identified inhibitors of equilibrative nucleoside transporter 1 (ENT1), a glycoprotein that mediates cellular uptake of nucleosides, from a small library screen of approved pharmacological agents as capable of augmenting oncolytic HSV infection. The ENT1 inhibitors, dilazep and dipyridamole, increased the spread and subsequent oncolytic ability of HSV by increasing ribonucleoside activity in many cancer cell lines but not in normal epithelial cells, an effect that required defective viral *ICP6*.

### Taking Over the Protein Synthesis Machine

While enhancing metabolic activity, suppressing antiviral signaling, and increasing nucleotide levels are all necessary and potentially rate-limiting steps for optimizing cancer growth or viral infection, the translation of proteins ultimately represents the final hurdle that ensures either cellular growth/division or the production of viral particle components. Dysregulation of translational control is one of the key events that promotes cellular transformation, and enhanced ribosome biogenesis, elevated levels of initiation factors, and changes in transcriptional repressors are found in a broad spectrum of cancers [69]. Since all eukaryotic viruses are fully dependent on host cell translational machinery to synthesize viral proteins, virus-host cell interactions that regulate translation, both globally and for specific mRNAs, contribute to the oncotropism of certain OVs (Figure 2). Generally, changes in the expression or availability of translational machinery components in cancer cells increase the overall rate of protein production (including viral polypeptides) [70],[71], and therefore enhance OV replication in tumours compared to normal tissues [72],[73].

Translation of most viral mRNA depends on the cellular cap-dependent program, where the rate-limiting initiation step represents a prime target for viral control. Viral dsRNA activates cellular protein kinase R (PKR), which inactivates the translation initiation factor eIF2 to limit host cell growth and initiate antiviral responses. As eIF2 phosphorylation dramatically reduces the efficiency and rate of viral translation, several viruses directly or indirectly prevent PKR activation. For example, HSV-1 encodes both US11, which directly inactivates PKR, and ICP34.5, which recruits cellular phosphatases to oppose PKR [74]. Similarly, VV encodes a PKR-binding protein (E3L) and a PKR pseudo-substrate (K3L) [74],[75]. There is evidence that eIF2 phosphorylation is dysregulated during tumourigenesis and, as a result, the inactivation of viral gene products involved in modulating PKR responses can generate an oncotropic virus. Most notably, a version of VSV expressing a catalytically inactive version of eIF2B-epsilon has a reduced ability to grow in benign cells compared to the parental VSV, but retains the ability to grow in malignant cells [76].

The mammalian target of rapamycin signalling complex 1 (mTORC1) is a master regulator of cell growth and metabolism; as such its activation promotes various anabolic processes in the cell, including protein biosynthesis. Notably, mTORC1 stimulates protein translation by inhibiting the translational repressor eIF4E-binding protein 1 (4EBP1) [77]. Herpes-, adeno-, and poxviruses encode viral products that ultimately inactivate 4EBP1, often through the mTORC1 pathway, and thus promote viral protein synthesis. For example, the HSV-1 protein kinase, US3, directly phosphorylates and inactivates 4EBP1 [78]. Since the mTORC1 pathway is almost always constitutively activated in malignant disease [79], US3-deficient HSV-1 mutants have a strong specificity for translationally active cancer cells [80]. The pharmacological manipulation of mTOR in combination with OVs has been explored using rapamycin, a well-tolerated and well-characterized mTOR inhibitor. Rapamycin and other “rapalogues” prevent the phosphorylation and activation of S6K and eIF4e, and retain the translational repressor, 4EBP1, in an active form. Rapamycin therefore hampers translation of both viral and cellular transcripts, but particularly negatively affects cellular mRNAs with complex secondary structures, such as those encoding the antiviral effectors, IFN and interferon regulatory factor 7 (IRF7), thereby favouring viral spread [81]–[84]. Inhibiting mTOR decreases activity of the downstream S6K, which in turn relieves its inhibitory effects on phosphatidylinositide 3-kinases (PI3K) and Akt signalling. For some viruses, such as myxoma virus (MYXV), increased activation of Akt improves replication and oncolytic activity in several mouse, rat, and human tumour models [85]–[87]. While systemic immunosuppressive effects of rapamycin or rapalogues also likely play a key role, such drugs have nevertheless been successfully used with several OVs, including adenovirus, HSV, VSV, MYXV, and VV, to improve their control of tumour progression [81],[86],[88]–[90].

Under conditions of cellular stress (e.g., hypoxia) where levels of eIF4E are low, tumours can also drive the expression of key malignancy-associated genes using a cap-independent (IRES-mediated) mechanism. Cap-independent translation often requires extremely high levels of eIF4G, which are typically found in transformed cells [91],[92]. Notably, several oncolytic picornaviruses (e.g., poliovirus and coxsackieviruses) lack the m7-cap required for normal translation, and thus encounter a strategic translational advantage in malignant cells [93]. Functional studies indicate that the rate of interaction between IRES and eIF4G may be an important factor that determines oncolytic poliovirus selectivity and enhanced propagation in certain tumour cells, such as glioblastoma [93]. In addition, recent studies showed that several noncanonical factors are recruited to bind the poliovirus IRES to modulate translation, and may play a role in cancer cell specificity [94].

### Riding the Tumour Cell Cycle

Dysregulation of the cell cycle to allow unchecked proliferation is a critically important step in tumourigenesis. Many of the key checkpoint proteins that normally regulate and restrict cell cycle progression, such as p53, RB, and myc, are actively disengaged, destabilising cells and priming them for further genetic mutations [95]. The successful replication of many wild-type viruses, including adenovirus, HSV, VV, MYXV, and reovirus, also requires effective targeting of central hubs of the proliferative signaling circuitry (Figure 2). While many viruses are capable of entering into and even initiating virus transcription in quiescent and terminally differentiated cells, the host cell must often enter the cell cycle to complete the viral replication cycle [96]–[99]. Viral proteins have therefore evolved to induce cell cycling, ensuring the activation of cellular biosynthetic machinery and mobilization of substrates necessary for the production of viral progeny. For example, the adenovirus E1A protein plays a key role in activating proliferation and cell cycle progression by binding and abrogating the activity of the retinoblastoma (RB) protein family [100],[101]. Since cancer cells almost uniformly downregulate such tumour suppressive proteins to release proliferation control, partial deletion of the *E1A* viral gene generates a specific oncolytic adenovirus [102]. MYXV and reovirus are also able to efficiently replicate and lyse a wide variety of human tumour cell lines with dysfunctional tumour suppressor genes, such as *RB* and *TP53* [103].

Increases in the frequency of mitogenic signals received through receptors such as epidermal growth factor receptor (EGFR) and its main downstream signaling transduction partners, PI3K/AKT and RAS/Mitogen-Activated Protein Kinase Kinase/Mitogen-Activated Protein Kinase (RAS/MEK/MAPK), also drive transformed or infected cell proliferation. Both herpes- and poxviruses activate the EGFR during their replication cycle [104],[105]. For example, VV encodes vaccinia growth factor (VGF), an early secreted protein that binds EGFR and conditions surrounding cells for subsequent viral infection [106]. Since EGFR is often constitutively activated in gliomas and carcinomas of the lung, colon, head and neck, pancreas, and breast [107], such tumours are rendered hypersensitive to VV replication. Numerous OVs, including MYXV, VV, coxsackievirus B3, and HSV-1, target the PI3K/AKT signal transduction pathway [85],[108]–[110]. For example, MYXV encodes the M-T5 host range factor, which induces the phosphorylation of cellular AKT and creates a growth-favourable environment for virus replication [111]. While mutant MXYV viruses lacking *M-T5* replicate poorly in most cells, tumour cells where AKT is constitutively activated are permissive to these viruses [112]. Several viruses also target the Ras signaling cascade. Replication of HSV-2 is facilitated by the viral *ICP10* gene–encoded serine/threonine protein kinase (PK) domain, which activates the Ras/MEK/MAPK pathway. Deletion of this PK domain converts HSV-2 into a potent oncolytic agent, exhibiting preferential replication in and lysis of tumour cells with a constitutively activated Ras signaling pathway [113]. Exploitation of the Ras cascade is also a critical step for reovirus particle uncoating, infectivity, and release, and Ras transformation is necessary to realize potent oncolytic effects [114],[115]. Activation of the Ras pathway has also been linked to repression of the antiviral response by interfering with PKR, Retinoic Inducible Gene I (RIG-I), and IFN signaling [46],[115].

## To Kill or Not to Kill: Balancing Apoptosis

The activation of the apoptotic pathway is a potent homeostatic tool for the early suppression of malignant cell outgrowth, while in infected cells, it represents a highly effective host response to curtail the infection cycle. Cellular changes that allow the avoidance of programmed cell death are essential for both tumour cell and virus host cell survival. Cancer cells employ multiple and diverse strategies to bypass the apoptotic death pathway normally induced by various stressors, and this evasive ability likely contributes to the survival and prolonged replication of naturally occurring or recombinant OVs. Yet while malignant apoptotic resistance supports optimal oncolytic virus growth, it can also compromise therapeutic efficacy, particularly given the importance of cytotoxic T cell–mediated killing of both infected and noninfected cancer cells for eliciting therapeutic responses following OV administration [116]–[119]. As such, a significant number of genetic strategies have aimed to improve direct and indirect cell killing upon OV infection. Multiple viruses encode viral proteins that target and regulate key steps in apoptotic pathways, balancing the maximum output of viral progeny with the necessity of keeping infected cells alive until successful transmission is achieved (Figure 2). For example, VV encodes several genes that modulate apoptosis, including *F1L* and *SPI-1* (*B22R*), which directly inhibit pro-apoptotic Bcl-2–like proteins and caspase activation, respectively. Deletion of these viral genes enhances tumour selectivity compared with wild-type VV [120]. The adenoviral *E1B-19K* gene product performs a similar function, blocking apoptosis by sequestering and inhibiting numerous pro-apoptotic Bcl-2–like proteins. Multiple lines of evidence indicate that *E1B-19K*-deficient adenoviruses selectively replicate and kill tumour cells *in vitro* and *in vivo*, with no effects in normal cells [121].

The loading of OVs with suicide transgenes has also been employed by several groups to enhance killing of both infected and uninfected tumour cells. Successful virally encoded suicide payloads include TNF-related apoptosis-inducing ligand (TRAIL) [122], Fas ligand (FasL) [123], yeast cytosine deaminase (in combination with 5-FC) [124], HSV thymidine kinase (in combination with gancyclovir) [125], *Drosophila melanogaster* multisubstrate deoxyribonucleoside kinase [126], uracil phosphoribosyltransferase (in combination with 5-FU) [127], carboxypeptidase G2 (in combination with ZD2767P) [128], and carboxylesterase (in combination with irinotecan) [129].

Drugs can also be used to increase virus-induced tumour cell death. For example, while HDIs impact the cellular IFN response (as discussed above), they also increase virus-induced apoptosis [58]. Another example comes from an elegant study by Mahoney *et al.*, who identified that knockdown of several proteins involved in the unfolded protein response significantly increased oncolytic Maraba virus–induced cell death following a genome-wide siRNA screen [130]. Knockdown of one of these genes, *IRE-1*, hampered the ability of cancer cells to cope with unfolded protein overload within the ER, effectively priming cancer but not benign cells to undergo virally induced apoptosis. A salicylaldehyde-based drug inhibitor of IRE-1 was subsequently synthesized and used to improve oncolytic Maraba efficacy in a resistant tumour model.

## Future Perspectives

With OV cancer therapeutics entering advanced-stage trials and showing clinical efficacy, strategies that further broaden OV targeting and replication capacity to address the heterogeneous nature of tumours and their associated vascular and stromal architecture will be extremely useful. Since such heterogeneity not only exists between patients but also within a given tumour/patient, where the metabolism, signal transduction, and antiviral states of cancer cells can be variably abnormal and, therefore, variably support OV replication, combinatorial strategies will be essential to promoting reliable tumour control and regression. Finally, continued efforts to identify components innate to the complex tumour microenvironment that promote OV replication will be critical to further improving OVs and developing new engineering strategies.
